# Erythritol Exacerbates DSS-Induced Colitis and Influences Behavioral Responses in Mice Through Gut Microbiota and Metabolic Alterations

**DOI:** 10.3390/microorganisms14081696

**Published:** 2026-08-02

**Authors:** Yingying Liu, Tian He, Wenle Liu, Limei Shao, Wei Lv, Linglong Ji, Ruihang Li, Haoran Nie, Qinghua Tan, Ling Liu

**Affiliations:** 1 Department of Gastroenterology and Hepatology, West China Hospital, Sichuan University, Chengdu 610041, China; yingying3957@stu.scu.edu.cn (Y.L.); hetian1@stu.scu.edu.cn (T.H.); liuwenle1@stu.scu.edu.cn (W.L.); m18267952034@163.com (L.S.); lvweieg@163.com (W.L.); 2019181620241@stu.scu.edu.cn (L.J.); 2020181620109@stu.scu.edu.cn (R.L.); 2021181620245@stu.scu.edu.cn (H.N.); tanqh2020@wchscu.cn (Q.T.); 2 The Center of Gerontology and Geriatrics, West China Hospital, Sichuan University, Chengdu 610041, China

**Keywords:** erythritol, DSS-induced colitis, gut microbiota, ferroptosis, tryptophan metabolism

## Abstract

Excessive consumption of ultra-processed foods (UPFs) containing non-nutritive sweeteners (NNS) has been implicated in inflammatory bowel disease (IBD), yet the effects of erythritol on intestinal inflammation remain poorly understood. In this study, we assessed NNS-containing UPF exposure in patients with IBD and healthy controls, and then investigated the effects of erythritol in a dextran sulfate sodium (DSS)-induced colitis. Food frequency questionnaire (FFQ) results showed that patients with IBD, particularly those with active disease, had a higher proportion of NNS-containing UPF categories among total UPFs consumed. In mice, erythritol administration exacerbated DSS-induced colitis, as evidenced by sustained body weight loss, increased disease activity index, impaired intestinal barrier integrity, and elevated inflammatory cytokine expression. Transcriptomic analysis revealed significant enrichment of ferroptosis-related pathways in the colonic mucosa of erythritol-treated colitic mice. Furthermore, erythritol markedly altered gut microbial composition, characterized by increased abundance of potentially pathogenic bacteria and enrichment of *Alistipes* sp. *CHKCI003*. Untargeted metabolomics demonstrated alteration of tryptophan metabolism. Consistently, erythritol aggravated depression-like behavior in DSS-induced mice. Collectively, these findings suggest that erythritol exacerbates experimental colitis and depression-like behavior, potentially through microbiota dysbiosis, ferroptosis-associated alterations, and disrupted tryptophan metabolism.

## 1. Introduction

Inflammatory bowel disease (IBD) is a chronic, non-specific, relapsing–remitting inflammatory intestinal disorder with unknown etiology, and has emerged as a pressing global public health challenge [1,2]. Multiple factors, including genetic susceptibility, environmental exposures, gut microbiota dysbiosis, and intestinal mucosal barrier dysfunction, have been implicated in the pathogenesis of IBD [3,4,5]. Among these factors, diet stands out as a particularly modifiable determinant of gut homeostasis [6]. Multiple large-scale prospective cohort studies have confirmed a significant positive association between high UPFs intake and an elevated risk of developing IBD [7,8]. Non-nutritive sweeteners (NNSs), core additive ingredients in UPFs, are widely used for their capacity to deliver sweet taste without calories, and their consumption has undergone explosive growth [9,10]. Recent evidence indicates that NNSs may disrupt the gut microbiota composition [11], impair intestinal mucosal barrier integrity [12], elevate pro-inflammatory cytokine levels [13], and activate inflammatory signaling pathways [14], thereby destabilizing the gut microenvironment and exacerbating gut inflammatory injury.

Erythritol is a four-carbon sugar alcohol used as a non-nutritive sweetener (NNS), with near-zero caloric value and a sweetness approximately 60–80% that of sucrose [15]. Approved as a food additive by multiple international food-safety authorities, it has become one of the fastest-growing NNSs worldwide [16,17]. After ingestion, 60–90% is rapidly absorbed in the small intestine and excreted unchanged in the urine, while the remaining fraction passes into the large intestine, where it becomes accessible to the gut microbiota for fermentation and metabolism [18]. Despite its widespread use and presumed safety, the effects of erythritol on the gut remain poorly defined and strikingly inconsistent, with reported outcomes ranging from harmful to protective. On the harmful side, erythritol has been shown to promote M1 macrophage polarization and elevate pro-inflammatory cytokines, thereby aggravating DSS-induced colitis [19], and elevated plasma erythritol has been clinically associated with the intestinal epithelial injury marker I-FABP (OR = 2.08, *p* = 0.004) [20]. Conversely, erythritol has been reported to reduce intestinal permeability in diabetic mice, suppress PI3K/AKT/NF-κB signaling and decrease *Desulfovibrio* abundance [21], and reinforce barrier function through goblet-cell hyperplasia and microbiota-derived, acetate-dependent stem-cell proliferation [22]. To date, studies on erythritol in the DSS-induced colitis model have focused predominantly on the acute phase of inflammation, with little attention to the recovery phase, and accompanying changes in the gut microbiota, metabolome, and transcriptome remain poorly characterized.

Patients with IBD endure considerable psychological burden [23]. Systematic reviews and meta-analyses have reported that anxiety symptoms occur in 32.1% IBD patients, while approximately a quarter (25.2%) experience depressive symptoms. Notably, both anxiety and depression are significantly more severe during active disease than during remission [24]. The gut–brain axis is considered one of the key mechanistic underpinnings of the elevated prevalence of anxiety and depressive disorders in IBD, although the precise regulatory pathways involved have yet to be fully elucidated [25]. Emerging evidence suggests that erythritol may participate in the modulation of emotional disturbances. Jiang et al. demonstrated that erythritol exacerbates anxiety-like behavior in mice with acute colitis [19]. Furthermore, abnormally elevated erythritol levels have been detected in brain tissue from patients with bipolar disorder [26] and in plasma samples from individuals with postpartum depression [27]. However, the precise effects of erythritol on anxiety and depression, as well as the molecular mechanisms through which it mediates emotional dysregulation, remain to be clearly defined.

Southwest China harbors a distinctive dietary culture, exemplified by Sichuan cuisine, one of the major culinary traditions in China. Building on these regional dietary characteristics, our prior research has shown that targeted dietary modulation can improve both symptoms and gut microbiota in patients with irritable bowel syndrome (IBS) [28]. In this study, we aimed to establish an IBD cohort and conduct a food frequency questionnaire (FFQ) to explore the intake patterns of nutrients, UPFs, and NNS-containing UPFs among IBD patients in this region. Using a dextran sulfate sodium (DSS)-induced murine colitis model, combined with multi-omics sequencing and behavioral assays, we systematically investigated the effects of erythritol on the gut microbiota, inflammatory responses, and anxiety- and depression-like behaviors in mice with colitis, and initially explored the underlying mechanisms. The findings are expected to provide important clinical guidance for erythritol consumption management and dietary intervention strategies in IBD patients.

## 2. Materials and Methods

### 2.1. Questionnaire Survey Design

From November 2022 to March 2024, patients with IBD were recruited from the outpatient clinics and inpatient wards of West China Hospital of Sichuan University, while healthy controls (HCs) were recruited from the health examination center. Written informed consent was obtained from all participants prior to enrollment. The FFQ was developed by the Department of Clinical Nutrition, based on the questionnaire (CNHS2002-D3) originally designed by the National Institute for Nutrition and Health, Chinese Center for Disease Control and Prevention, with modifications according to dietary habits in the Sichuan region. To minimize recall bias and measurement error, 1:1 scale food models calibrated to actual weights, together with real commercial UPF, were provided on-site during FFQ administration. Participants could inspect and handle these items to accurately report their food consumption frequency and portion sizes over the one-month period prior to the survey. Open-ended items were also included in the questionnaire to capture unlisted food items and mitigate underreporting. All FFQs were administered by experienced clinical nutrition physicians. According to the *Chinese Food Composition Table*, *Standard Edition* (6th ed.), the average daily intake over the preceding month was calculated for total energy, protein, fat, carbohydrates, insoluble dietary fiber, total UPF weight, energy derived from UPFs, proportion of energy intake contributed by UPFs, categories of NNS-containing UPFs, total categories of UPFs consumed, and category proportion of NNS-containing UPFs among all UPFs. Nutritional information for UPFs was obtained from the nutrition facts labels on food packaging and supplemented using the Mint Health application (v12.4.1). The presence of NNSs was determined based on the ingredient lists provided on food packaging.

The Inflammatory Bowel Disease Questionnaire (IBDQ) consists of a total of 32 questions in four sections: bowel symptoms, systemic symptoms, emotional function, and social function. The higher the score, the better the health status or quality of life [29]. This study was approved by the Biomedical Ethics Review Committee of West China Hospital, Sichuan University (1718 in 2022), and was also registered online in the Chinese Clinical Trial Registry (ChiCTR2200066486).

### 2.2. Participant Recruitment

#### 2.2.1. Inclusion and Exclusion Criteria for Patients with IBD

Inclusion criteria were as follows: (1) provision of written informed consent and (2) a confirmed clinical diagnosis of IBD. Exclusion criteria were as follows: (1) refusal to provide informed consent; (2) presence of metabolic diseases requiring dietary control, including diabetes mellitus, hypertension, gout, or hyperlipidemia; (3) recent surgery resulting in changes in dietary habits; (4) current long-term enteral or parenteral nutritional support due to disease exacerbation, malignancy, or other conditions; and (5) inability to understand or complete the questionnaire.

#### 2.2.2. Inclusion and Exclusion Criteria for Healthy Controls

Inclusion criteria were as follows: (1) provision of written informed consent and (2) regular eating habits and a healthy lifestyle. Exclusion criteria were as follows: (1) refusal to provide informed consent; (2) major dietary modifications due to hypertension, diabetes mellitus, gout, hyperlipidemia, major cardiovascular or cerebrovascular diseases, fatty liver disease, gastrointestinal diseases, or other medical conditions; (3) substantial recent changes in dietary habits caused by surgery, major life events, or other reasons; and (4) inability to understand or complete the questionnaire.

### 2.3. Animals

Five-week-old male C57BL/6 mice were purchased from Sichuan Viton Lihua Laboratory Animal Technology Co., Ltd. (Chengdu, China). and housed under specific pathogen-free conditions at the Laboratory Animal Center of the Frontiers Science Center for Disease-Related Molecular Network, Sichuan University. Mice were maintained under controlled temperature and humidity with a 12 h light/dark cycle. All mice were acclimated for 7 days before experiments and had free access to standard chow and sterile water during this period. All animal procedures were approved by the Animal Ethics Committee of West China Hospital, Sichuan University (20230824003).

### 2.4. DSS-Induced Acute Colitis

Mice were randomly assigned to four groups: control, erythritol (ERY), DSS-induced colitis (DSS), and erythritol plus DSS-induced colitis (ERY+DSS). All core phenotypic indicators (body weight, DAI score, colon length, and survival rate) were statistically analyzed using all eligible mice in each group. Sterile drinking water served as the vehicle for all groups. The control group received water alone throughout the experiment, and the ERY group received 5% (*w*/*v*) erythritol throughout [30]. The DSS group received plain water for the first 3 weeks, then 2.5% (*w*/*v*) DSS (M.W: 36,000–50,000, MP Biomedicals Inc., Solon, OH, USA) for 7 days, after which DSS was withdrawn. Mice in the ERY+DSS group received 5% (*w*/*v*) erythritol in sterile drinking water throughout the experiment, with 2.5% (*w*/*v*) DSS added for 7 days after the first 3 weeks. During DSS administration, body weight, stool consistency, and fecal occult blood were recorded daily to assess the disease activity index (DAI) [31]. The total DAI score for each mouse ranged from 0 to 12, according to the scoring criteria shown in Supplementary Table S1. Mice were euthanized 3 days after DSS withdrawal. The entire colon was immediately dissected and measured for length. Proximal and distal colon tissues were fixed in 4% paraformaldehyde. Colonic mucosa was gently scraped, and cecal and colonic fecal samples were collected, immediately snap-frozen in liquid nitrogen, and stored at −80 °C until further analysis.

### 2.5. Histological Analysis and Special Staining

Proximal and distal colon tissues were paraffin-embedded and sectioned at 4 μm for subsequent staining. Hematoxylin and eosin (H&E) staining was performed, and histological scoring was conducted according to the criteria described in our previous study [32,33]. Alcian blue–periodic acid–Schiff (AB–PAS) staining was used to visualize the intestinal mucus barrier. Immunohistochemistry was performed using a universal mouse/rabbit IHC detection kit (PK10006, Proteintech, Wuhan, China) according to the manufacturer’s instructions. After antigen retrieval, sections were incubated with primary antibodies against 4-hydroxynonenal (4-HNE; 4 hydroxynonenal rabbit pAb, bs-6313R, 1:100; Bioss, Beijing, China), acyl-CoA synthetase long-chain family member 4 (ACSL4; ACSL4 rabbit mAb, A20414, 1:200; ABclonal, Wuhan, China), ferritin light chain (FTL; ferritin light-chain rabbit mAb, A11241, 1:200; ABclonal, Wuhan, China) and ferritin heavy chain (FTH; ferritin heavy-chain PolymAb, A25458-PM, 1:200; ABclonal, Wuhan, China) overnight at 4 °C or for 2 h at room temperature. After rewarming to room temperature and three washes in PBS, sections were incubated with the secondary antibody (HRP-conjugated goat anti-rabbit/mouse IgG polymer, PK10006, Proteintech, Wuhan, China) for 30 min at room temperature, then dehydrated, mounted, and examined under a light microscope. For immunofluorescence, antigen-retrieved sections were incubated with anti-ZO-1 (ZO1 rabbit pAb, YN1410, 1:200; Immunoway, Suzhou, China) and anti-occludin (occludin polyclonal antibody, 27260-1-AP, 1:800; Proteintech, Wuhan, China) overnight at 4 °C. After washing in PBST, sections were incubated with a fluorescent red secondary antibody (donkey anti-rabbit IgG H&L, BF555 conjugated, bs-0295D-BF555, 1:500; Bioss, Beijing, China) for 1 h at room temperature, mounted, and observed under a fluorescence microscope.

### 2.6. Reverse-Transcription Quantitative Real-Time Polymerase Chain Reaction (RT-qPCR)

To determine the mRNA levels of cytokines, colonic mucosal tissue was homogenized and total RNA was extracted using the Foregene Animal Total RNA Isolation Kit (Foregene, Chengdu, China). cDNA was synthesized from 500 ng RNA using the First-Strand cDNA Synthesis Master Premix (for qPCR) RT EasyTM II kit. Quantitative PCR was then performed with 2× SYBR Green Master Mix to measure the pro-inflammatory cytokines interleukin 1β (IL-1β) and tumor necrosis factor α (TNF-α). β-actin or GAPDH mRNA served as the internal reference for normalization, and relative gene expression was calculated using the 2^−ΔΔCt^ method. Primers were synthesized by Tsingke Biotechnology (Beijing, China), with sequences listed in Supplementary Table S2.

### 2.7. 16S rRNA Sequencing

Microbial genomic DNA was extracted from mouse cecal fecal samples using the TGuide S96 Magnetic Bead Stool Genomic DNA Extraction Kit (Tiangen Biotech Co., Ltd., Beijing, China) according to the manufacturer’s instructions. Full-length 16S rRNA genes were amplified by PCR under the following conditions: initial denaturation at 95 °C for 2 min; followed by 22 cycles of 98 °C for 10 s, 55 °C for 30 s, and 72 °C for 10 min 30 s; with a final extension at 72 °C for 4 min. PCR products were examined by agarose gel electrophoresis, and qualifying samples were selected for subsequent analysis. Purified PCR products were subjected to library construction, sequencing, and quality control on the PacBio platform by Beijing Biomarker Technology Co., Ltd. (Beijing, China). α diversity was evaluated using the Chao1 and Shannon indices, while β diversity was assessed by principal coordinate analysis (PCoA) and analyzed using PERMANOVA. Linear discriminant analysis effect size (LEfSe) was performed to identify differentially abundant taxa among groups at the genus and species levels, with a linear discriminant analysis (LDA) score threshold of >3.

### 2.8. RNA Sequencing

Total RNA was extracted from mouse colonic mucosa using TRIzol Reagent (Life Technologies, Carlsbad, CA, USA). RNA concentration, purity, and integrity were subsequently assessed. Sequencing libraries were prepared using the Hieff NGS Ultima Dual-mode mRNA Library Prep Kit (Yeasen Biotechnology Co., Ltd., Shanghai, China) according to the manufacturer’s instructions, followed by library fragment purification and quality evaluation. Qualified libraries were sequenced on the Illumina NovaSeq platform, and raw sequencing data were further processed using the BMKCloud bioinformatics analysis platform (www.biocloud.net). Transcriptome sequencing was performed by Beijing Biomarker Technology Co., Ltd. (Beijing, China). Differentially expressed genes (DEGs) among groups were identified using DESeq2 software (version 1.30.1). Genes with a fold change (FC) > 2 and a false-discovery rate (FDR) < 0.05 were considered significantly differentially expressed. Functional enrichment and clustering analyses of DEGs were performed based on the Gene Ontology (GO) and Kyoto Encyclopedia of Genes and Genomes (KEGG) databases to identify key differential genes and associated biological pathways. Gene set enrichment analysis (GSEA) was subsequently conducted.

### 2.9. Untargeted Metabolomic Analysis

Metabolite extraction and analysis of colonic fecal samples were performed by Beijing Biomarker Technology Co., Ltd. (Beijing, China). Untargeted metabolomic profiling in both positive- and negative-ion modes was conducted using a Waters Acquity I-Class PLUS ultrahigh-performance liquid chromatograph coupled with a Waters Xevo G2-XS QTOF high-resolution mass spectrometer (both from Waters, Shanghai, China). Metabolite peaks from raw data were identified based on the METLIN database and the self-built database of Biomarker Technology. Differential metabolites and their associated functions were annotated using the Human Metabolome Database (HMDB) and KEGG database. Principal component analysis (PCA) and orthogonal projections to latent structures—discriminant analysis (OPLS—DA) were performed to evaluate differences in metabolite distribution and variation among groups. Differential metabolites were screened based on FC, variable importance in projection (VIP), and *p* values, with thresholds set at FC ≥ 1.5, VIP ≥ 1, and *p* < 0.05. KEGG pathway analysis was subsequently performed for functional annotation and enrichment analysis of differential metabolites.

### 2.10. Behavioral Tests

#### 2.10.1. Elevated Plus Maze (EPM)

The EPM test was performed on day 5 of DSS administration. The EPM apparatus consisted of two open arms (300 mm × 50 mm), two closed arms (300 mm × 50 mm × 160 mm), and a central platform (160 mm × 160 mm), elevated approximately 70 cm above the floor. At the beginning of each test, mice were placed in the central area facing an open arm and allowed to freely explore the apparatus for 5 min, while behavior was recorded using a camera positioned above the maze. The percentage of open arm entries [open arm entries/(open arm entries + closed arm entries)], the percentage of time spent in the open arms [time spent in open arms/(time spent in open arms + closed arm time)], and total travel distance were analyzed. The procedures were performed as previously described [34].

#### 2.10.2. Open Field Test (OFT)

The OFT was conducted as previously described [35]. Briefly, mice were placed in an open-top apparatus (495 mm × 495 mm × 350 mm) divided into central and peripheral zones. At the beginning of each trial, mice were placed in the center area and allowed to freely explore for 5 min while behavior was recorded using a camera mounted above the apparatus. The total travel distance, number of entries into the central zone, and time spent in the central zone were analyzed.

#### 2.10.3. Tail Suspension Test (TST)

The TST was performed according to previously published methods [36]. Mice were suspended by the tail using adhesive tape in a 200 mm × 200 mm × 250 mm chamber, with the head positioned approximately 20 cm above the floor. Mouse behavior, including alternating periods of struggling and immobility, was recorded for 6 min using a camera positioned in front of the apparatus. Total immobility time was measured, with longer immobility times indicating greater depression-like behavior.

All behavioral tests were conducted in a quiet and dimly lit room. The apparatus was cleaned and disinfected with 75% ethanol after each trial to eliminate residual odors and excreta. Behavioral data were analyzed using EthoVision XT (version 17.5, Noldus Information Technology, Leesburg, VA, USA).

### 2.11. Statistical Analysis

Data are presented as means ± standard deviation (SD) and as medians (interquartile range, IQR) for non-normally distributed variables. Statistical analyses and data visualization were performed using GraphPad Prism (version 9.5.1, GraphPad Software, San Diego, CA, USA). Categorical variables were analyzed using the chi-squared test or Fisher’s exact test. Normality was assessed using the Shapiro–Wilk test. Depending on the data distribution and experimental design, differences between groups were evaluated using Student’s *t*-test, Mann–Whitney *U* test, one-way or two-way analysis of variance (ANOVA), or the Kruskal–Wallis test, followed by Tukey’s or Bonferroni’s post hoc test where appropriate. Analysis of covariance (ANCOVA) was used for normally distributed data requiring adjustment for confounding factors, while nonparametric ANCOVA was applied for non-normally distributed data. Survival rates were compared using the log-rank test. Correlations between two variables were assessed using Spearman’s rank correlation analysis. For visualization, scatterplots were overlaid with a simple linear regression line and its 95% confidence interval. A two-sided *p* < 0.05 was considered statistically significant.

## 3. Results

### 3.1. IBD Patients Exhibited Increased Exposure to NNS-Containing UPFs

A total of 44 patients with ulcerative colitis (UC), 57 patients with Crohn’s disease (CD), and 106 HCs were initially recruited in this study. After applying the inclusion and exclusion criteria, 35 UC patients, 50 CD patients, and 99 HCs with complete dietary questionnaires and IBDQ data were included in the final analysis. Baseline characteristics of the participants are summarized in Table 1. Compared with HCs, CD patients were younger, had lower body mass index (BMI), and showed a higher proportion of males.

After adjustment for age, sex, and BMI, no significant differences were observed among HCs, UC, and CD groups in total daily energy intake, protein intake, fat intake, carbohydrate intake, total UPF consumption by weight, UPF-derived energy intake, or the proportion of energy derived from UPFs (all *p* > 0.05, Table 1). However, CD patients exhibited significantly lower insoluble dietary fiber intake compared with HCs (*p* = 0.003).

Notably, although both UC (*p* = 0.001) and CD patients (*p* < 0.001) consumed significantly fewer total categories of UPFs than HCs, and CD patients consumed fewer categories of NNS-containing UPFs than HCs (*p* = 0.003), the proportion of NNS-containing UPF categories relative to total UPF categories was significantly higher in IBD patients than in HCs (*p* = 0.002). In particular, UC patients showed a significantly higher proportion of NNS-containing UPFs compared with HCs (*p* < 0.05). Moreover, the intake of UPFs by weight, UPF-derived energy intake, the proportion of energy derived from UPFs, and the proportion of NNS-containing UPF categories all exhibited a trend of CD > UC > HCs, although these differences did not reach statistical significance.

According to disease activity status at the time of questionnaire completion, IBD patients were further stratified into active disease (*n* = 55) and remission groups (*n* = 30, Table 2). No significant differences in age, sex, or BMI were observed between the two groups (*p* > 0.05). Similarly, no significant differences were found in carbohydrate intake, insoluble dietary fiber intake, UPF consumption by weight, UPF-derived energy intake, proportion of energy derived from UPFs, categories of NNS-containing UPFs, or total categories of UPFs between active and remission groups (all *p* > 0.05). However, patients with active IBD exhibited a higher proportion of NNS-containing UPF categories relative to total UPF categories than patients in remission (75.0% vs. 64.59%, *p* = 0.05, Table 2), suggesting a greater exposure tendency to NNS-containing UPFs in active IBD. Furthermore, patients with active IBD showed significantly lower daily energy (*p* = 0.01), protein (*p* = 0.001), and fat (*p* = 0.019) intake compared with patients in remission.

Consistently, IBDQ scores were significantly lower in patients with active disease than in those in remission (Supplementary Table S3).

### 3.2. Erythritol Aggravates DSS-Induced Colitis and Impairs Intestinal Barrier

**Figure 1:**
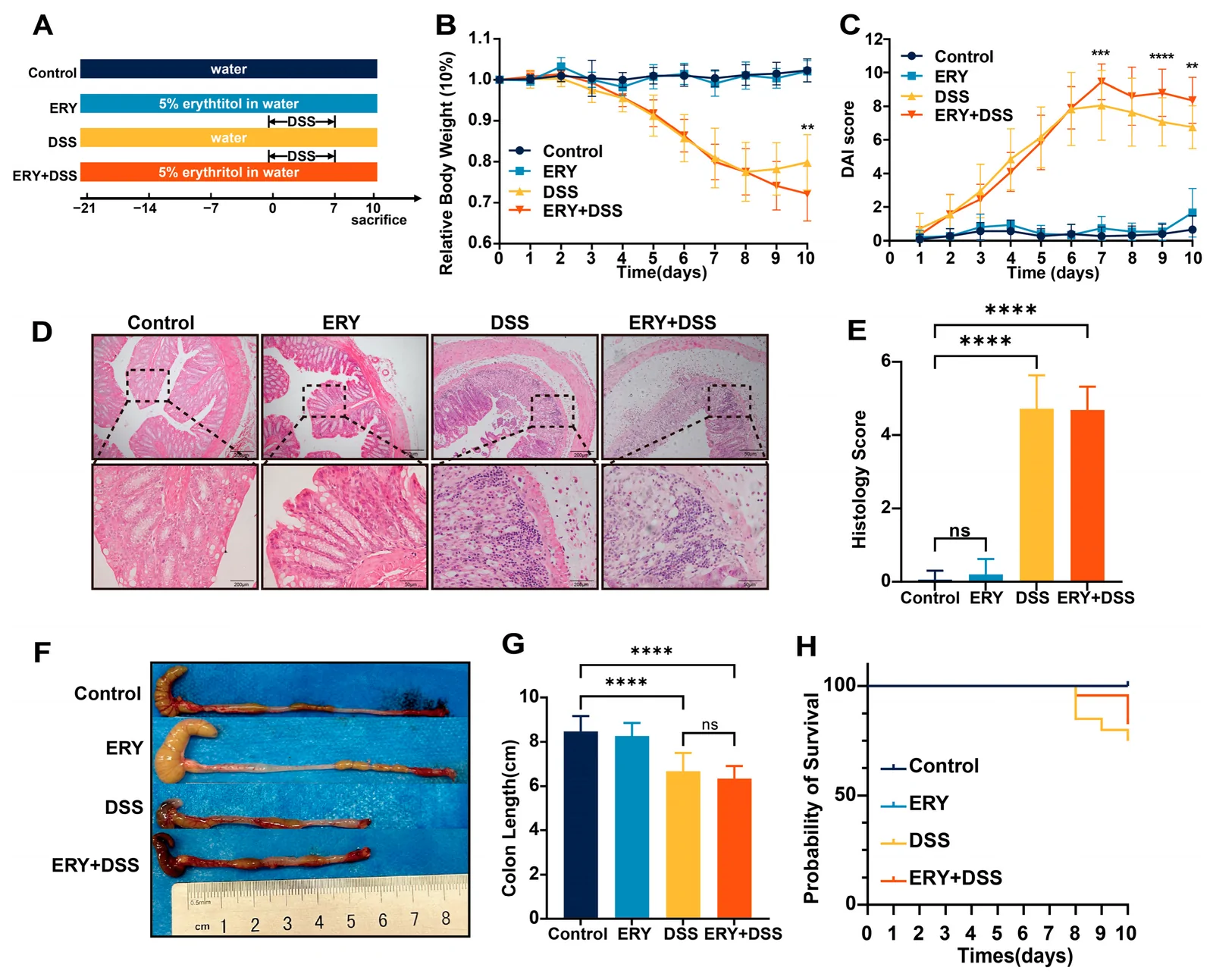
Erythritol exacerbated DSS-induced colitis in mice. (**A**) Experimental design of erythritol administration and DSS-induced colitis model. (**B**) Changes in body weight during the experimental period. (**C**) Disease activity index (DAI) scores. (**D**) Representative hematoxylin and eosin (H&E)-stained colon sections from each group (100× and 400×). Scale bars, 200 μm (above) and 50 μm (below). (**E**) Histological scores of colonic tissues. (**F**) Representative images of colons from each group. (**G**) Colon length measurements. (**H**) Survival curves of mice during the experimental period. These results present data from 15–23 mice per group; histological score were assessed for 10 mice per group. ** *p* < 0.01; *** *p* < 0.001; **** *p* < 0.0001; ns, not significant.

To investigate the impact of erythritol on DSS-induced acute colitis, mice were treated according to the experimental design shown in Figure 1A. During DSS administration, both the DSS and ERY+DSS groups exhibited progressive body weight loss and increased DAI scores. Notably, following DSS withdrawal, mice in the DSS group gradually recovered, as evidenced by body weight regain and reduced DAI scores. In contrast, mice in the ERY+DSS group continued to lose weight and maintained higher DAI scores, which became significantly greater than those of the DSS group on day 10 (Figure 1B,C). Histopathological analysis revealed marked crypt destruction, inflammatory cell infiltration, and mucosal edema in both DSS-treated groups (Figure 1D). Histological scores and colon length were comparable between the DSS and ERY+DSS groups (Figure 1E–G). Nevertheless, both groups displayed significantly higher mortality than the control group (Figure 1H).

**Figure 2:**
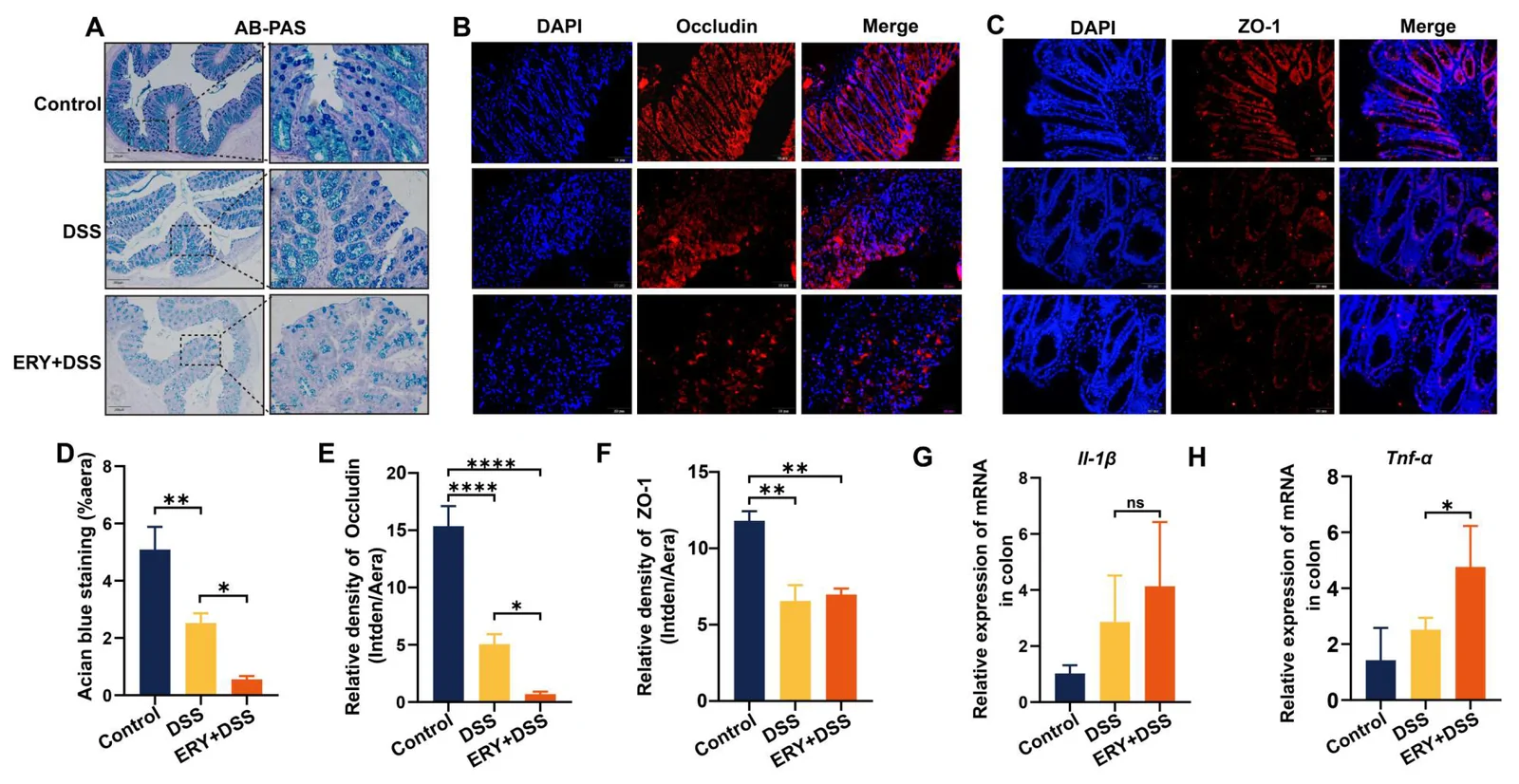
Erythritol aggravated intestinal barrier damage and inflammatory responses. (**A**) Representative Alcian blue–periodic acid–Schiff (AB–PAS) staining of colon sections (100× and 400×). Scale bars, 200 μm (above) and 50 μm (below). (**B**) Representative immunofluorescence staining of occludin (400×). Scale bar = 50 μm. (**C**) Representative immunofluorescence staining of ZO-1 (400×). Scale bar = 50 μm. Nuclei were stained with DAPI (blue); occludin and ZO-1 were visualized in red. (**D**) Quantification of the AB–PAS-positive area. (**E**) Quantification of the occludin-positive area. (**F**) Quantification of the ZO-1-positive area. (**G**) Relative mRNA expression of *Il-1β* in colonic tissues. (**H**) Relative mRNA expression of *Tnf-α* in colonic tissues. *n* = 6 mice per group for staining assays and qPCR. * *p* < 0.05; ** *p* < 0.01; **** *p* < 0.0001; ns, not significant.

To further assess intestinal barrier integrity, AB–PAS staining and tight junction protein expression were evaluated. Compared with the control group, DSS treatment markedly reduced goblet-cell abundance, and this reduction was more pronounced in the ERY+DSS group (Figure 2A,D). Immunofluorescence staining demonstrated decreased expression of the tight junction proteins occludin and ZO-1 following DSS exposure relative to the control group. Compared with the DSS group, the ERY+DSS group exhibited a further reduction in occludin expression, whereas ZO-1 expression showed no significant difference between the two groups (Figure 2B,C,E,F). These findings indicate that erythritol exacerbates DSS-induced impairment of the intestinal mucosal barrier. Consistent with the aggravated intestinal injury, colonic mRNA expression of the pro-inflammatory cytokine TNF-α was significantly higher in the ERY+DSS group than in the DSS group, whereas IL-1β expression did not differ significantly between the two groups (Figure 2G,H), suggesting an enhanced inflammatory response following erythritol exposure.

### 3.3. Erythritol Promotes Ferroptosis-Associated Alterations in the Colon of DSS-Induced Colitic Mice

**Figure 3:**
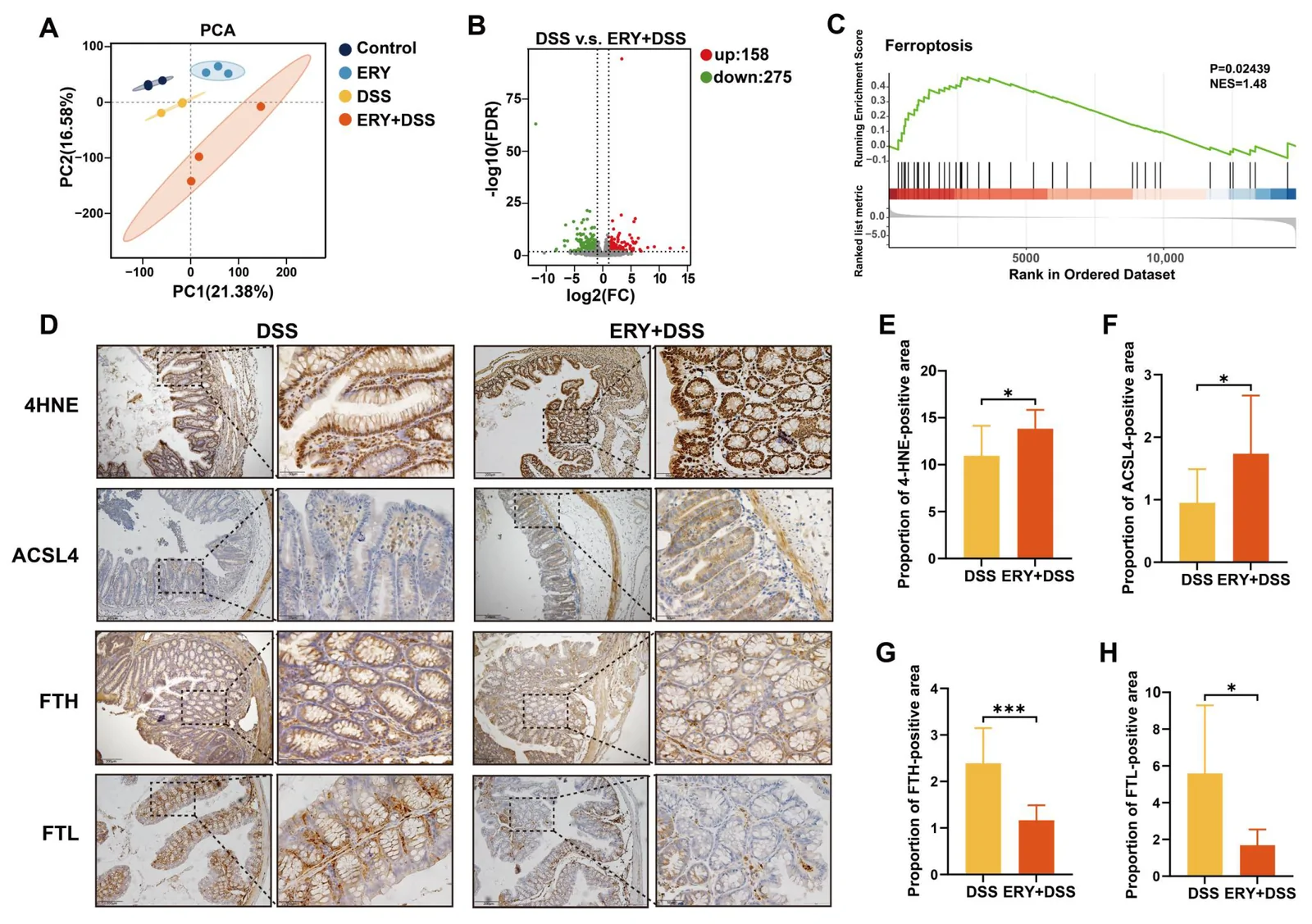
Transcriptomic analysis revealed ferroptosis-related alterations. (**A**) PCA of colonic transcriptomic profiles (*n* = 3 per group). (**B**) Volcano plot showing differentially expressed genes between the DSS and ERY+DSS groups. (**C**) GSEA showing enrichment of the ferroptosis pathway in the ERY+DSS group. (**D**) Representative immunohistochemical staining of ferroptosis-related markers (4-HNE, ACSL4, FTH, and FTL) in colonic tissues (100× and 400×). Scale bars, 200 μm (right) and 50 μm (left). Immunohistochemical staining showed brown positive signals, and nuclei were counterstained blue with hematoxylin. (**E**) Quantification of the 4-HNE-positive area. (**F**) Quantification of the ACSL4-positive area. (**G**) Quantification of the FTH-positive area. (**H**) Quantification of the FTL-positive area. *n* = 6 mice per group for immunohistochemical quantification. * *p* < 0.05; *** *p* < 0.001.

To investigate the effects of erythritol on host gene expression under inflammatory conditions, transcriptomic profiling was performed on colonic tissues from each group. PCA demonstrated clear separation among groups, indicating that both DSS treatment and erythritol intervention markedly altered the colonic transcriptional landscape (Figure 3A). Compared with the DSS group, 158 genes were upregulated and 275 genes were downregulated in the ERY+DSS group (Figure 3B). KEGG enrichment analysis revealed that compared with the DSS group, the pathways upregulated in the ERY+DSS group included glutathione metabolism, drug metabolism–cytochrome P450, circadian rhythm, pentose and glucuronate interconversions, glyoxylate and dicarboxylate metabolism, galactose metabolism, mineral absorption and glycerolipid metabolism (Supplementary Figure S1A). Relative to the DSS group, pathways including the intestinal immune network for IgA production, the B-cell receptor signaling pathway and natural killer cell–mediated cytotoxicity were significantly downregulated in the ERY+DSS group (Supplementary Figure S1B). GSEA further revealed significant enrichment of the ferroptosis pathway in the ERY+DSS group (Figure 3C), suggesting that erythritol may promote ferroptosis-associated molecular events during colonic inflammation. Consistent with the GSEA results, immunohistochemical staining showed that the ERY+DSS group exhibited significantly increased 4-HNE- and ACSL4-positive areas compared with the DSS group (Figure 3D–F). In contrast, the positive areas of the iron storage proteins FTH and FTL were significantly decreased in the ERY+DSS group (Figure 3D,G,H). These findings further support that erythritol supplementation promotes ferroptosis-associated alterations in the colon of DSS-induced colitic mice.

### 3.4. ERY Altered Gut Microbiota Composition

**Figure 4:**
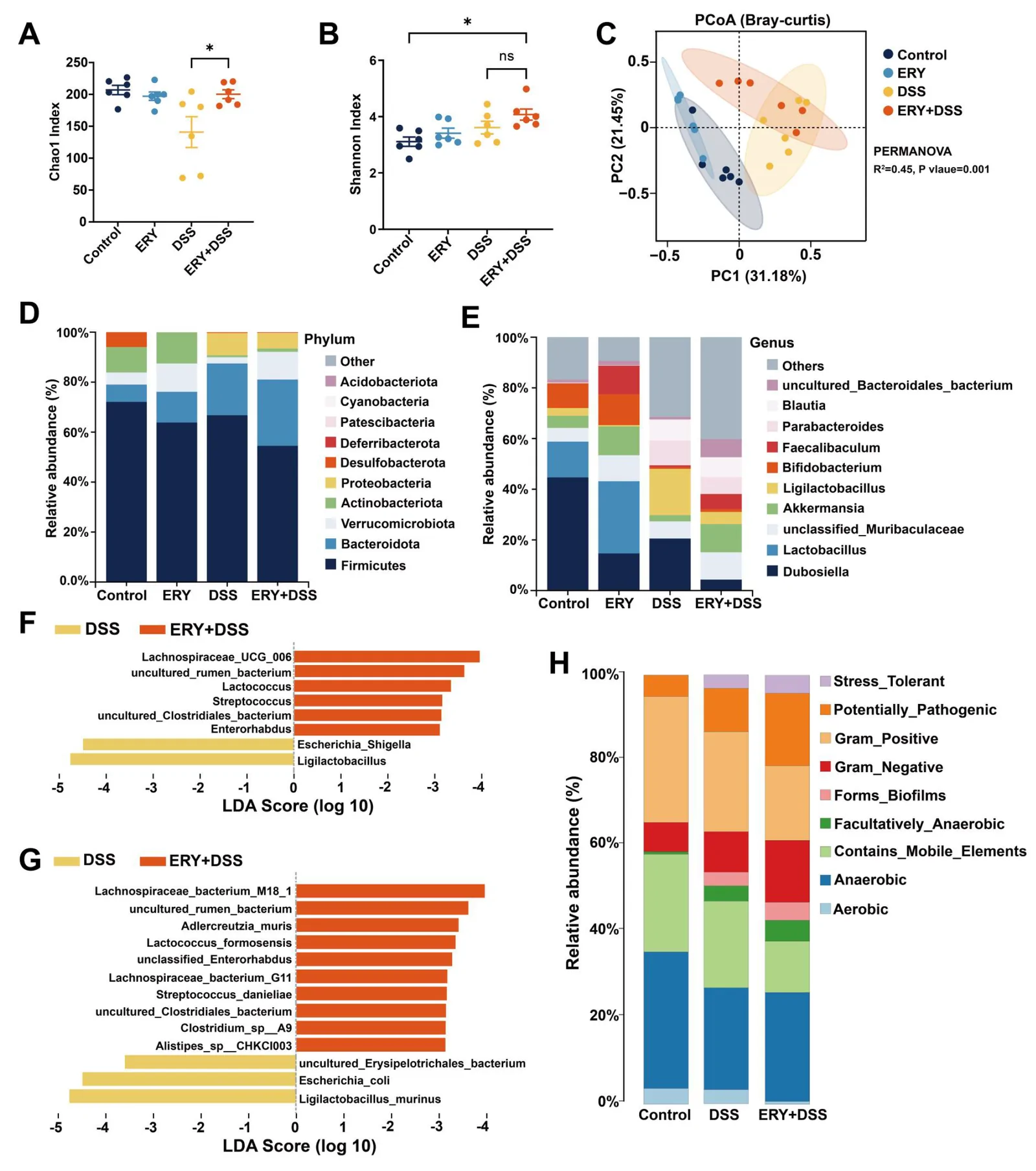
Erythritol reshaped the gut microbial community in DSS-induced colitis (*n* = 6 per group). (**A**) Chao1 index. (**B**) Shannon index. (**C**) PCoA showing β diversity among the four groups in total. (**D**) Relative abundance of the top 10 bacterial phyla. (**E**) Relative abundance of the top 10 bacterial genera. (**F**) LDA scores of differentially abundant genera identified by LEfSe analysis between the DSS and ERY+DSS groups (LDA > 3). (**G**) LDA scores of differentially abundant species identified by LEfSe analysis between the DSS and ERY+DSS groups (LDA > 3). (**H**) BugBase-based microbial phenotype prediction in the control, DSS, and ERY+DSS groups. * *p* > 0.05. ns, not significant.

To investigate the effects of erythritol on gut microbial alterations during DSS-induced colitis, 16S rRNA amplicon sequencing was performed using cecal contents. Compared with the DSS group, ERY+DSS mice exhibited a significantly increased Chao1 index, whereas no significant difference was observed in the Shannon index (Figure 4A,B), suggesting altered microbial richness following erythritol intervention under inflammatory conditions. PCoA based on Bray–Curtis distances demonstrated distinct microbial community clustering among groups (PERMANOVA, *p* = 0.001; Figure 4C), indicating that both DSS and erythritol treatment reshaped gut microbial structure. At the phylum level, ERY treatment further reduced the relative abundance of *Firmicutes* while increasing *Bacteroidota* and *Verrucomicrobiota* in DSS-treated mice (Figure 4D). At the genus level, ERY+DSS mice showed decreased relative abundance of *Dubosiella* and *Ligilactobacillus*, along with increased *Akkermansia* abundance compared with DSS mice (Figure 4E). LEfSe analysis further identified distinct microbial signatures among groups. Compared with DSS mice, ERY+DSS mice were enriched in several taxa, including *Lachnospiraceae_UCG_006*, *Lactococcus*, *Streptococcus*, *Enterorhabdus*, and *Alistipes*_sp.__*CHKCI003*, whereas *Escherichia_Shigella* and *Ligilactobacillus_murinus* were reduced (Figure 4F,G). BugBase phenotype prediction analysis suggested that ERY treatment increased the relative abundance of Gram-negative and potentially pathogenic bacteria under DSS conditions (Figure 4H).

### 3.5. Metabolomic Analysis Revealed Alterations in Tryptophan Metabolism

**Figure 5:**
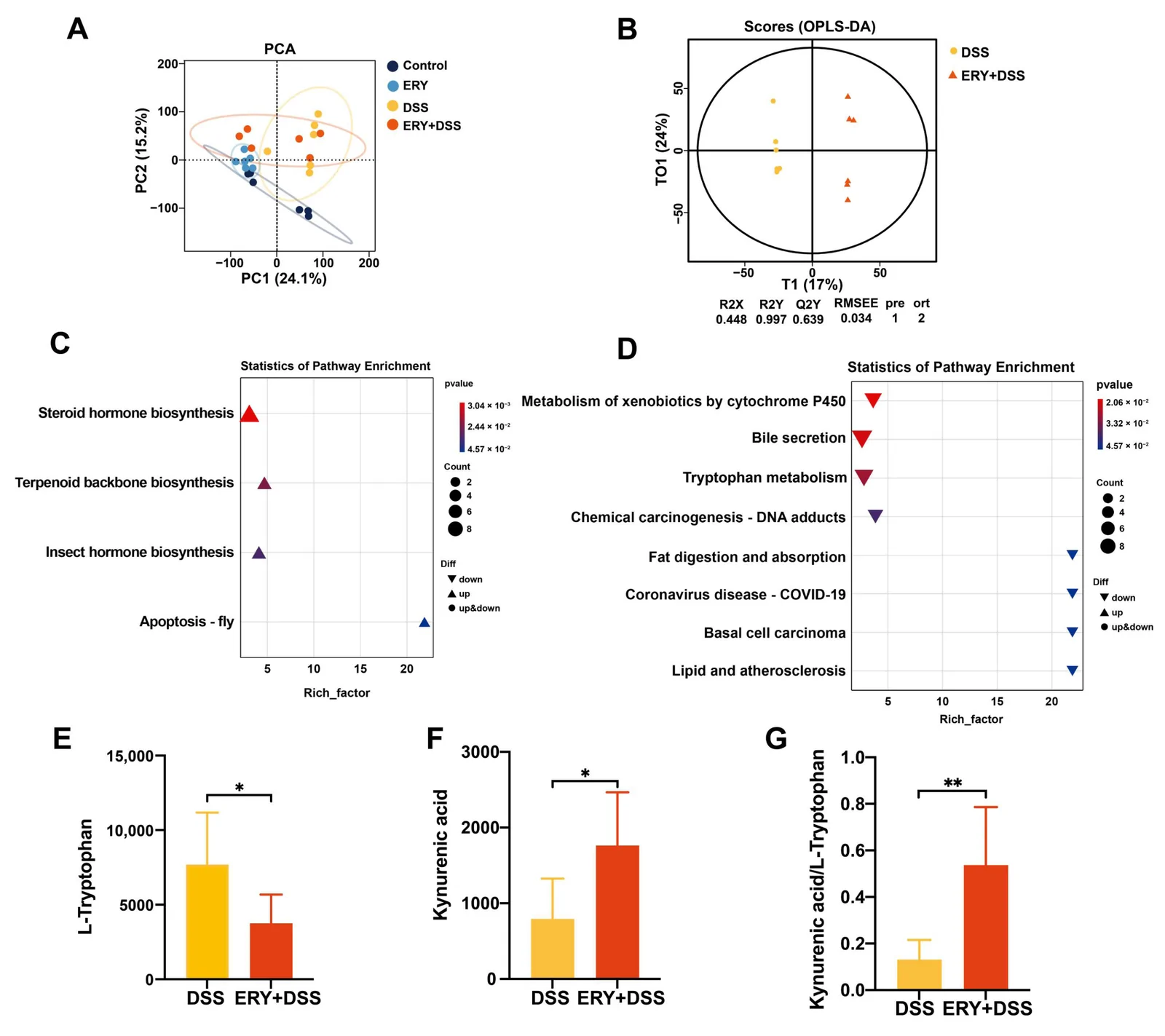
Metabolomic profiling revealed alterations in tryptophan metabolism (*n* = 6 per group). (**A**) PCA of fecal metabolomic profiles. (**B**) OPLS—DA comparing the DSS and ERY+DSS groups. (**C**) KEGG enrichment analysis of metabolites significantly increased in the ERY+DSS group relative to the DSS group. (**D**) KEGG enrichment analysis of metabolites significantly decreased in the ERY+DSS group relative to the DSS group. (**E**) L-tryptophan levels in the DSS and ERY+DSS groups. (**F**) Kynurenine levels in the DSS and ERY+DSS groups. (**G**) Kynurenine-to-L-tryptophan ratio. * *p* < 0.05; ** *p* < 0.01.

To investigate the impact of erythritol on intestinal metabolism under inflammatory conditions, untargeted metabolomic profiling was performed using colonic contents. PCA revealed a partial separation of metabolic profiles among the four experimental groups (Figure 5A). OPLS—DA further demonstrated a clear distinction between the DSS and ERY+DSS groups (R^2^X = 0.448, R^2^Y = 0.997, Q^2^ = 0.639), indicating that erythritol intervention markedly altered the intestinal metabolic profile in DSS-induced colitis mice (Figure 5B). KEGG pathway enrichment analysis showed that upregulated differential metabolites in the ERY+DSS group were primarily enriched in steroid hormone biosynthesis and terpenoid backbone biosynthesis pathways (Figure 5C). The KEGG term “insect hormone biosynthesis” in Figure 5C was retained as an enrichment output, but should be interpreted cautiously, as it likely reflects sterol-related metabolite annotation rather than actual insect hormone biosynthesis in mice. In contrast, downregulated metabolites were mainly enriched in metabolism of xenobiotics by cytochrome P450, bile secretion, fat digestion and absorption, and tryptophan metabolism pathways (Figure 5D). Compared with the DSS group, the ERY+DSS group showed a significant decrease in L-tryptophan levels (Figure 5E), whereas kynurenine abundance (Figure 5F) and the kynurenine-to-L-tryptophan ratio (Figure 5G) were significantly elevated.

### 3.6. Erythritol Selectively Altered TST Immobility in DSS-Induced Colitis Mice

**Figure 6:**
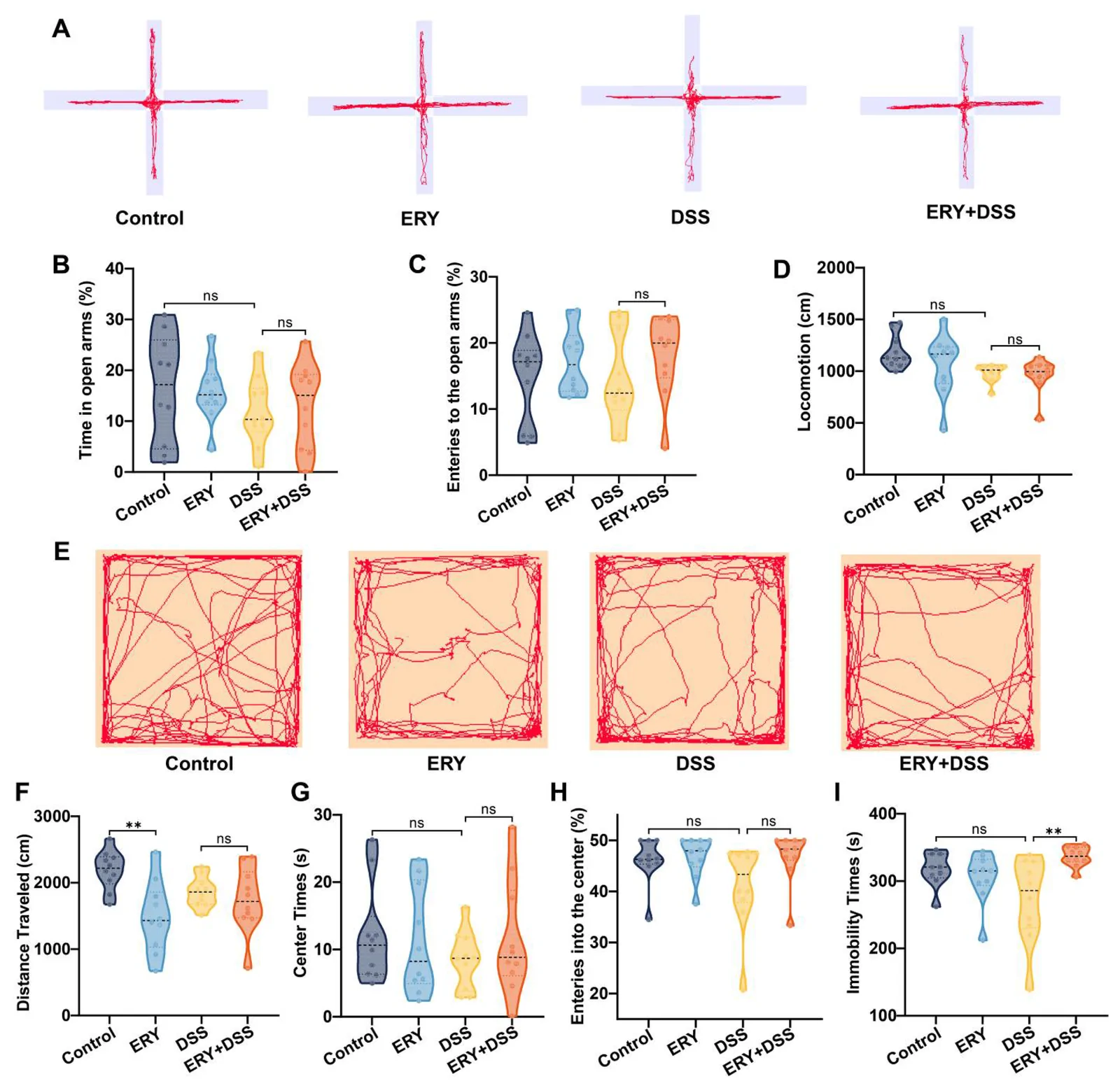
Erythritol aggravated depression-like behavior (*n* = 10 per group). (**A**) Representative movement trajectories in the elevated plus maze (EPM) test. (**B**) Percentage of time spent in the open arms. (**C**) Percentage of entries into the open arms. (**D**) Total distance traveled in the EPM test. (**E**) Representative movement trajectories in the open field test (OFT). (**F**) Total distance traveled in the OFT. (**G**) Time spent in the center zone during the OFT. (**H**) Percentage of entries into the center zone during the OFT. (**I**) Immobility time in the tail suspension test (TST). ** *p* < 0.01. ns, not significant.

To evaluate the effects of erythritol intervention on anxiety- and depression-like behaviors in DSS-induced colitis mice, the elevated plus maze test, open field test, and tail suspension test were performed. In the elevated plus maze test, no significant differences were observed among the four groups in the percentage of open arm entries, percentage of time spent in the open arms, or total travel distance (Figure 6A–D). In the open field test, compared with the control group, ERY-treated mice showed no significant changes in the time spent in the center area or the percentage of entries into the center area, whereas the total travel distance was significantly reduced. Moreover, no significant differences were observed between the DSS and ERY+DSS groups in the time spent in the center area, percentage of entries into the center area, or total travel distance (Figure 6E–H). In the TST, the immobility time of DSS-treated mice was not significantly different from that of control mice (Figure 6I), suggesting that acute DSS-induced colitis did not induce obvious depression-like behavioral changes under our experimental conditions. However, the immobility time in the ERY+DSS group was significantly increased compared with the DSS group, while it was not significantly different from the control group (Figure 6I). These results indicate that erythritol supplementation increased the TST-related immobility response under DSS-treated conditions to some extent, but did not result in a significant aggravation of depression-like behavior.

### 3.7. Correlations Between Alistipes sp. CHKCI003, Tryptophan Metabolites, and Depression-like Behavior

**Figure 7:**
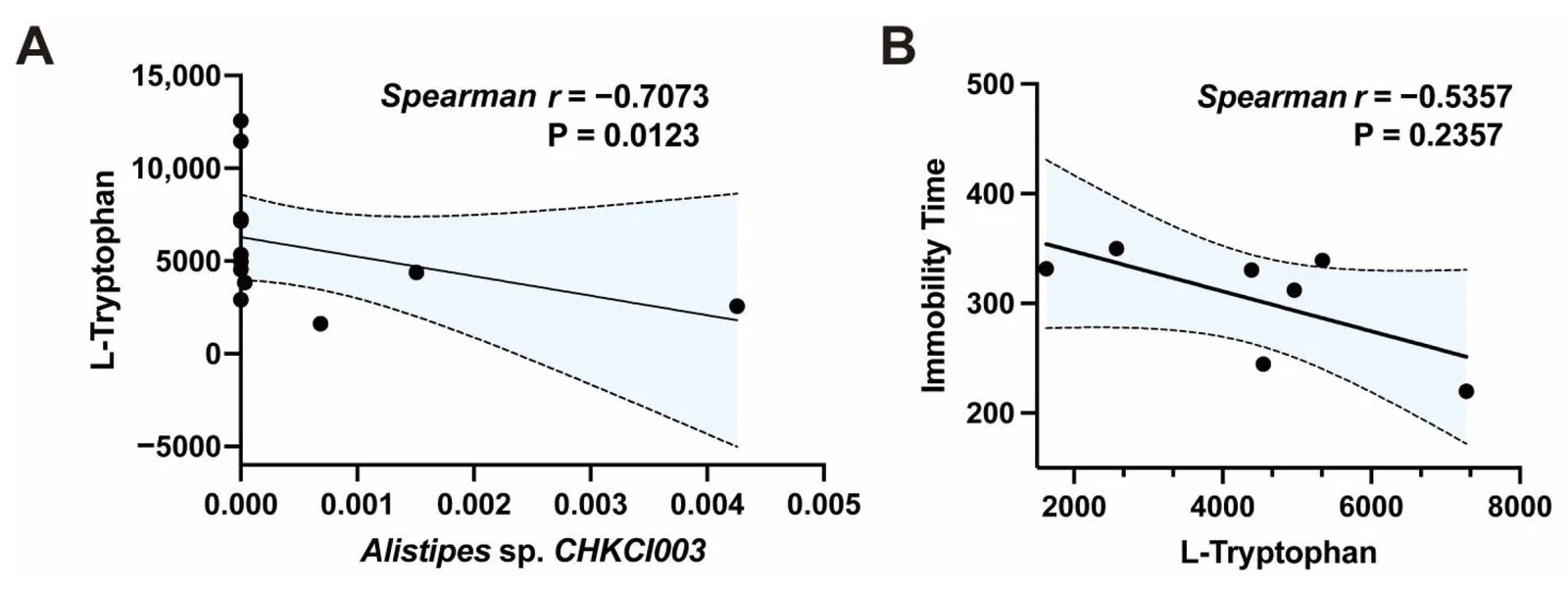
Correlations among *Alistipes* sp. *CHKCI003*, tryptophan metabolites, and depression-like behavior. (**A**) Spearman correlation analysis between L-tryptophan levels and the abundance of *Alistipes* sp. *CHKCI003* (*n* = 10). (**B**) Spearman correlation analysis between immobility time and L-tryptophan levels (*n* = 7). Solid lines represent the simple linear regression fit, and shaded areas indicate the corresponding 95% confidence intervals.

Previous studies have reported that the genus *Alistipes* is closely associated with tryptophan metabolism [37]. As the abundance of *Alistipes* sp. *CHKCI003* was significantly increased in the ERY+DSS group, we further examined the correlations between this species and L-tryptophan. Spearman correlation analysis revealed a significant negative correlation between L-tryptophan levels and the abundance of *Alistipes* sp. *CHKCI003* (*r* = −0.7073, *p* = 0.0123; Figure 7A). In addition, the immobility time in the TST was negatively correlated with L-tryptophan levels, although the correlation did not reach statistical significance (*r* = −0.5457, *p* = 0.2357; Figure 7B).

## 4. Discussion

By combining a dietary survey of patients with IBD and DSS-induced experimental colitis, this study preliminarily investigated the effects of erythritol exposure on intestinal inflammation, the gut microbiota, and metabolism. We found that: (1) patients with IBD showed a higher exposure risk to UPFs containing NNSs; (2) erythritol aggravated DSS-induced colitis and intestinal mucosal barrier damage; (3) transcriptomic and histological results suggested that erythritol promoted ferroptosis-related changes; (4) erythritol significantly altered the composition of the gut microbiota and the metabolic profile, affecting the tryptophan metabolism pathway; and (5) these changes were accompanied by aggravated depression-like behavior in mice.

In recent years, a growing body of epidemiological evidence has shown that the intake of UPFs is closely associated with an increased risk of IBD [38,39]. A study using an FFQ to assess the relationship between UPFs intake and disease activity in patients with IBD revealed that UC patients with higher UPFs consumption experienced a greater number of active disease episodes [40]. NNSs, a class of food additives widely used in UPFs, have been shown in previous studies to disturb intestinal homeostasis by reshaping the gut microbiota, disrupting the intestinal mucosal barrier, and modulating host immune responses [41]. A survey of the dietary habits of patients with IBD by Basson et al. found that compared with HCs, patients with IBD consumed artificial sweeteners and low-calorie foods more frequently, whereas their intake frequency of sucrose was relatively lower, suggesting that patients with IBD may be more inclined to choose sweetener-containing products [42]. Consistent with these studies, our study found that although the total number of UPF categories consumed by patients with IBD was lower than that of healthy controls, the proportion of NNS-containing UPF categories among all UPF categories was significantly higher, and this proportion was further increased in patients with active IBD (Table 1 and Table 2). To our knowledge, this is the first study to combine a clinical cohort with an FFQ to characterize the intake patterns of UPFs, and particularly NNS-containing UPFs, among patients with IBD in the Sichuan cuisine region of western China.

Erythritol, a naturally occurring sugar alcohol with near-zero caloric content and a negligible effect on blood glucose, has been widely used in the food industry and has rapidly gained consumer favor [43,44]. However, a growing number of studies in recent years have shown that in addition to potentially increasing the risk of cardiovascular events [45], erythritol may also be involved in the regulation of inflammatory responses and oxidative stress. Previous studies have found that erythritol can induce oxidative stress and inflammatory responses in zebrafish embryos and affect organ development [46]. In addition, in THP-1-derived macrophages, erythritol can promote M1 polarization, increase reactive oxygen species (ROS) production, and activate the Akt signaling pathway, suggesting that it may have pro-inflammatory potential [47]. To date, research on erythritol and intestinal inflammation remains limited. Jiang et al. reported that erythritol can aggravate DSS-induced colitis in mice by promoting macrophage infiltration and M1 polarization and upregulating the expression of pro-inflammatory cytokines [19]. Our study further supports the detrimental effect of erythritol on intestinal inflammation. Compared with the DSS group, mice in the ERY+DSS group exhibited more severe body weight loss, higher DAI scores, more pronounced intestinal mucosal barrier damage, and higher TNF-α expression levels (Figure 1 and Figure 2). Notably, unlike previous studies that mainly focused on the active phase of the disease, we observed that mice treated with erythritol continued to show body weight loss and higher DAI scores during the recovery phase after DSS withdrawal (Figure 1B,C), suggesting that erythritol may not only aggravate inflammatory injury but also delay intestinal mucosal repair and disease recovery. It should also be noted that a study have reported that erythritol can attenuate high-fat diet-induced inflammation of the small intestine [30]. However, we consider that this finding does not contradict our results. High-fat diet-induced metabolic inflammation and DSS-induced acute colitis differ markedly in their pathogenesis, immune response characteristics, and patterns of intestinal mucosal injury [48,49]; therefore, erythritol may exert different biological effects under different pathological conditions.

In fact, erythritol alone was not sufficient to induce overt intestinal injury in our experimental condition (Figure 1). We speculate that the effects of erythritol on intestinal inflammation may be inflammation state-dependent: they are mainly amplified in the context of preexisting colitis or barrier damage, whereas erythritol does not produce detectable intestinal injury under healthy conditions. DSS-induced colitis disrupts epithelial tight junctions and increases intestinal permeability, which may represent the “first hit” that primes the intestinal environment [50]. Under this condition, erythritol exposure may act as a “second hit” by further modulating gut microbial composition, metabolic profiles, and host transcriptional responses, including ferroptosis-related alterations, thereby amplifying inflammatory responses and exacerbating tissue injury.

Ferroptosis is a form of programmed cell death characterized by the iron-dependent accumulation of lipid peroxidation, and its occurrence is jointly regulated by iron homeostasis, lipid metabolism, and the cellular antioxidant system [51,52]. In recent years, increasing evidence has shown that ferroptosis is involved in the development and progression of IBD [53]. Previous studies have found that the expression of various ferroptosis-related genes is abnormal in the intestinal mucosa of patients with IBD, accompanied by elevated lipid ROS levels [54]. In our study, we observed that erythritol treatment significantly altered the transcriptomic profile of colonic tissue. GSEA showed that compared with the DSS group, the ferroptosis pathway was significantly enriched in the ERY+DSS group (Figure 3C). To further verify this finding, we examined vital ferroptosis-related markers. The results showed that the expression of ACSL4, a key enzyme in lipid peroxidation [55], was significantly increased in the ERY+DSS group (Figure 3D,F), and the lipid peroxidation product 4-HNE [56] showed an increasing trend (Figure 3D,E); meanwhile, the expression of FTH and FTL, which are involved in intracellular iron storage [57], was further decreased (Figure 3D,G,H). The increase in ACSL4 suggests that polyunsaturated fatty acids in the cell membrane are more susceptible to peroxidation, whereas the decrease in FTH and FTL may lead to the accumulation of free iron, thereby aggravating oxidative damage. We speculate that erythritol may enhance lipid peroxidation and disrupt iron homeostasis, thereby increasing the susceptibility of the inflamed intestine to ferroptosis and contributing to the aggravation of colitis.

The gut microbiota is considered an important bridge linking dietary factors, intestinal inflammation, and host immune regulation [58]. In this study, we found that compared with the DSS group, the ERY+DSS group showed increased α diversity and marked changes in microbial composition (Figure 4A–C). Analysis at the phylum level showed that erythritol exposure further promoted the expansion of *Bacteroidetes* and *Proteobacteria* while reducing the relative abundance of *Firmicutes* and *Actinobacteria*, resulting in a decreased *Firmicutes*/*Bacteroidetes* ratio (Figure 4D). This change is consistent with the typical features of gut microbiota dysbiosis in patients with IBD [59]. Previous studies have shown that the commensal microbiota can maintain intestinal homeostasis by competitively inhibiting pathogen colonization, promoting mucus layer formation, and regulating adaptive immune responses [60,61]. In this study, the abundance of potentially beneficial bacteria such as *Dubosiella*, *Lactobacillus*, and *Ligilactobacillus* was reduced in the ERY+DSS group (Figure 4E), whereas BugBase analysis further revealed an increase in Gram-negative and potentially pathogenic bacteria (Figure 4H), suggesting that erythritol may promote a shift of the gut microbiota toward a more pro-inflammatory ecological structure, thereby contributing to the aggravation of colitis.

Notably, LEfSe analysis revealed that the abundance of *Alistipes* sp. *CHKCI003* was significantly increased in the ERY+DSS group (Figure 4G). *Alistipes*, an indole-producing gut bacterium, has been reported to be associated with depressive symptoms and stress exposure [37]. As a key mediator of the gut–brain axis, the gut microbiota is closely linked to the development of depression [62]. Previous studies have shown that *Alistipes* is significantly enriched in the feces of patients with major depressive disorder compared with healthy controls [63,64]. It has been reported that the potential mechanisms by which *Alistipes* contributes to depression may be related to its involvement in tryptophan metabolism and its reduction of serotonin levels [65]. Moreover, tryptophan supplementation has been shown to suppress the intestinal colonization of *Alistipes inops* and to ameliorate depression-like behaviors in mice [66].

Tryptophan is metabolized mainly through three pathways: the kynurenine, serotonin (5-hydroxytryptamine, 5-HT), and indole pathways [67]. In mammals, more than 90% of ingested tryptophan is metabolized via the kynurenine pathway, with the remainder being converted into serotonin and indole derivatives [67], thereby exerting substantial effects on the physiological and pathological processes of IBD, IBS, metabolic syndrome, and neuropsychiatric alterations—particularly anxiety, depression, and autism [68]. Tryptophan metabolism is closely associated with depression: under the influence of pro-inflammatory cytokines, the activity of key kynurenine-pathway enzymes is excessively activated, driving metabolic flux toward the kynurenine pathway; this increases neurotoxic metabolites and neuroinflammation while reducing serotonin production, thereby aggravating depression [69,70]. In our study, KEGG enrichment analysis showed that the tryptophan metabolism pathway was significantly altered (Figure 5D). Compared with the DSS group, the ERY+DSS group exhibited a significant decrease in intestinal L-tryptophan levels and a significant increase in kynurenine and the kynurenine/L-tryptophan ratio (Figure 5E–G), suggesting that erythritol may aggravate intestinal inflammation and thereby promote a shift of tryptophan metabolism toward the kynurenine pathway, with a corresponding reduction in serotonin-pathway metabolism. Spearman correlation analysis further revealed that the decreased L-tryptophan level was significantly negatively correlated with the increased abundance of *Alistipes* sp. *CHKCI003* (Figure 7A), and the immobility time in the tail suspension test showed a negative correlation trend with L-tryptophan levels (Figure 7B). These findings suggest a potential association among erythritol-induced gut microbial alterations, disrupted tryptophan metabolism, and increased TST immobility under DSS-treated conditions. EPM and OFT mainly assess anxiety-like behavior, exploratory activity, and locomotor performance, whereas TST reflects immobility responses under acute inescapable stress [71]. Notably, acute DSS treatment did not induce significant anxiety-like or depression-like behavioral changes in our study, and erythritol supplementation did not further elicit consistent anxiety-like alterations in DSS-induced colitis mice as assessed by the EPM and OFT (Figure 6). Although the ERY+DSS group showed a significantly longer immobility time than the DSS group in the TST, this parameter was not significantly different from that of the control group (Figure 6I). Therefore, erythritol may alter TST-related behavioral responses in mice with DSS-induced colitis, though whether this alteration represents an aggravation of depression-like behavior requires further investigation.

This study has several limitations. First, the FFQ used in this study could not quantify NNS intake. As erythritol currently has no established acceptable daily intake (ADI) and its added amount is not disclosed or standardized across manufacturers, the actual amount of erythritol consumed could not be determined. Additionally, differential recall bias may exist, as patients with active IBD and those in remission may differ in their attention to UPF and overall dietary intake due to different disease statuses. Future studies using more objective dietary assessment methods, such as repeated 24 h dietary recalls or circulating/urinary erythritol biomarkers, are warranted to validate and quantify this association. Second, although we observed a significant negative correlation between the abundance of *Alistipes* sp. *CHKCI003* and L-tryptophan levels, this finding does not establish causality. Whether erythritol-induced gut microbiota alterations directly lead to disrupted tryptophan metabolism and behavioral changes remains to be further validated in future mechanistic studies such as using fecal microbiota transplantation, germ-free or antibiotic-treated mouse models, and functional validation of specific bacteria. Third, although our transcriptomic analysis and immunohistochemical staining implicated ferroptosis-associated alterations in erythritol-exacerbated DSS colitis, these findings remain mainly observational. Functional validation, such as the use of ferroptosis inhibitors including ferrostatin 1, will be important in future mechanistic studies to determine whether ferroptosis causally contributes to the aggravating effect of erythritol on DSS-induced colitis. The specific mechanisms by which erythritol engages ferroptosis also require further investigation.

## 5. Conclusions

This study integrated clinical diet surveys, DSS-induced colitis models, behavioral tests, and multi-omics analyses to evaluate the effects of erythritol on intestinal inflammation. Clinically, IBD patients, particularly those with active disease, exhibited a higher proportion of NNS-containing UPFs, suggesting a potential association between exposure to NNS-containing UPFs and disease activity. Our experimental results indicate that erythritol may exacerbate DSS-induced colitis via ferroptosis and that enrichment of *Alistipes* sp. *CHKCI003* may disrupt tryptophan metabolism, thereby inducing TST-related behavioral abnormalities. Collectively, our findings underscore that IBD patients should be more cautious about the consumption of NNS-containing UPFs. Future studies will focus on elucidating the specific molecular mechanisms by which erythritol modulates ferroptosis to aggravate colitis and how this specific bacterium influences tryptophan metabolism and behavioral responses.

## Figures and Tables

**Table 1:** Baseline characteristics and dietary assessment of healthy controls and IBD patients.

	HCs (*n* = 99)	UC (*n* = 35)	CD (*n* = 50)	*p* Value
Age (years)	36.00 (30.00, 44.00)	39.00 (30.00, 52.00)	31.00 (23.00, 38.50)	0.003
Male (*n*%)	58 (58.6%)	14 (40.0%)	14 (28.6%)	0.002
BMI (kg/m^2^)	22.53 ± 0.31	20.88 ± 0.58	19.34 ± 0.39	<0.001
Energy, Kcal/d	1445.84 (1162.00, 1948.00)	1606.23 (1106.00, 1908.86)	1610.11 (1207.55, 2134.02)	0.879
Protein, g/d	48.46 (34.09, 60.00)	45.69 (30.19, 51.02)	44.31 (32.03, 61.38)	0.621
Fat, g/d	65.92 ± 2.97	64.17 ± 5.75	65.71 ± 4.15	0.821
Carbohydrates, g/d	180.69 (141.00, 247.65)	179.30 (147.04, 273.96)	210.45 (138.17, 327.81)	0.512
Insoluble dietary fiber, g/d	7.00 (4.63, 9.57)	4.78 (2.38, 7.52)	3.98 (2.57, 6.60) **	0.002
Weight of UPFs, g/d	9.67 (3.33, 24.33)	10.29 (4.29, 17.90)	13.15 (4.59, 47.50)	0.538
Energy from UPFs, Kcal/d	37.42 (9.36, 80.00)	42.62 (20.83, 76.08)	46.85 (17.70, 122.05)	0.378
Percentage of energy derived from UPFs, (%)	2.59 (0.61, 5.78)	2.75 (0.73, 5.69)	3.07 (1.24, 9.32)	0.435
Categories of NNS-containing UPFs, (*n*)	4.00 (2.00, 5.00)	3.00 (1.00, 4.00)	3.00 (1.00, 4.00) **	0.006
Total categories of UPFs, (*n*)	6.00 (4.00, 9.00)	4.00 (2.00, 6.00) *	4.00 (2.00, 6.00) **	<0.001
Percentage of NNS-containing UPF categories, (%)	55.56 (45.45, 71.43)	70.00 (62.50, 100.00) *	71.43 (50.00, 100.00)	0.002

HCs: healthy controls; BMI: body mass index; UPFs: ultra-processed foods; Data with normal distribution are presented as means ± standard deviation (SD), whereas non-normally distributed data are presented as medians (P25, P75). *p* values indicate statistical significance among the three groups. * Significant difference between healthy controls and UC patients; ** significant difference between healthy controls and CD patients.

**Table 2:** Baseline characteristics and dietary assessment according to disease activity in patients with IBD.

	IBD in Remission (*n* = 30)	Active IBD (*n* = 55)	*p* Value
Age (years)	31.50 (26.25, 40.00)	34.00 (27.00, 45.00)	0.262
Male (*n*%)	11 (36.70%)	18 (32.70%)	0.714
BMI (kg/m^2^)	20.06 ± 0.40	19.93 ± 3.55	0.835
Energy, Kcal/d	1752.33 (1539.20, 2229.52)	1390.15 (985.51, 1918.29)	0.010
Protein, g/d	49.54 (42.20, 75.84)	40.80 (23.79, 49.30)	0.001
Fat, g/d	75.70 ± 5.24	59.28 ± 4.21	0.019
Carbohydrates, g/d	230.34 (183.45, 334.68)	176.80 (133.31, 282.07)	0.053
Insoluble dietary fiber, g/d	4.90 (2.93, 7.93)	4.00 (2.20, 6.59)	0.316
Weight of ultra-processed foods (UPFs), g/d	10.86 (4.33, 25.10)	12.00 (5.00, 38.00)	0.581
Energy from UPFs, Kcal/d	44.83 (17.64, 86.77)	42.62 (19.30, 120.44)	0.568
Percentage of energy derived from UPFs, (%)	2.13 (0.93, 3.88)	3.67 (1.02, 9.35)	0.141
Categories of NNS-containing UPFs, (*n*)	2.50 (1.00, 4.00)	3.00 (1.00, 4.00)	0.369
Total categories of UPFs, (*n*)	4.00 (2.00, 5.00)	4.00 (2.00, 6.00)	0.908
Percentage of NNS-containing UPF categories, (%)	64.59 (50.00, 80.00)	75.00 (62.50, 100.00)	0.050

## Data Availability

All data are available in the main text or the Supplementary Materials. The raw 16S rRNA gene sequencing and transcriptome (RNA-seq) data have been deposited in the NCBI Sequence Read Archive (SRA) under BioProject accession number PRJNA1478140 (accessed on 28 June 2026). The untargeted metabolomic data have been deposited in the MetaboLights repository under accession number MTBLS14768 (accessed on 28 June 2026). Additional data related to this article are available from the corresponding author upon reasonable request.

## Supplementary Materials

The following supporting information can be downloaded at https://www.mdpi.com/article/10.3390/microorganisms14081696/s1. Figure S1: KEGG pathway enrichment analysis of differentially expressed genes between the DSS and ERY+DSS groups; Table S1: Disease activity index; Table S2: Summary Table of Primer Sequences; Table S3: IBDQ scores of active IBD and IBD in remission.

## Abbreviations

The following abbreviations are used in this manuscript.

UPFs	ultra-processed foods
NNS	non-nutritive sweeteners
IBD	inflammatory bowel disease
DSS	dextran sulfate sodium
FFQ	food frequency questionnaire
CD	Crohn’s disease
UC	Ulcerative colitis
SCFA	short-chain fatty acid
IBS	irritable bowel syndrome
HCs	healthy controls
IBDQ	Inflammatory Bowel Disease Questionnaire
ERY	Erythritol
DAI	Disease activity index
H&E	Hematoxylin and eosin
AB–PAS	Alcian blue–periodic acid–Schiff
4-HNE	4-hydroxynonenal
ACSL4	Acyl-CoA synthetase long-chain family member 4
FTL	ferritin light chain
FTH	ferritin heavy chain
RT-qPCR	reverse-transcription quantitative real-time polymerase chain reaction
TNF-α	tumor necrosis factor-α
IL-1β	interleukin-1β
PCoA	principal coordinate analysis
LEfSe	linear discriminant analysis effect size
LDA	linear discriminant analysis
DEGs	differentially expressed genes
FC	fold change
FDR	false-discovery rate
GO	Gene Ontology
KEGG	Kyoto Encyclopedia of Genes and Genomes
GSEA	gene set enrichment analysis
HMDB	Human Metabolome Database
PCA	principal component analysis
OPLS—DA	orthogonal projections to latent structures—discriminant analysis
VIP	variable importance in projection
EPM	elevated plus maze
OFT	open field test
TST	tail suspension test
SD	standard deviation
ANOVA	analysis of variance
ANCOVA	analysis of covariance
BMI	body mass index
ROS	reactive oxygen species
5-HT	5-hydroxytryptamine
ADI	acceptable daily intake
