# Supplementary material for: Erythritol Exacerbates DSS-Induced Colitis and Influences Behavioral Responses in Mice Through Gut Microbiota and Metabolic Alterations
Source: Microorganisms. 2026 Aug 2;14(8):1696. doi: 10.3390/microorganisms14081696 (PMC13515666; doi:10.3390/microorganisms14081696)
Supplement: Supplementary file 1 [file microorganisms-14-01696-s001.zip › microorganisms-4435728-supplementary.pdf]

Supplementary Table S1. Disease activity index.

score	Weight loss (%)	Stool consistency	Bloody stool
0	0	Normal	-
1	1-5%	-	+
2	5-10%	Loose stool	++
3	10-20%	-	+++
4	＞20%	Diarrhea	++++

Supplementary Table S2. Summary Table of Primer Sequences.

Gene	Forward primer	Reverse primer
β-actin	AAGGCCAACCGTGAAAAGAT	GTGGTACGACCAGAGGCATAC
TNF-α	GGTGCCTATGTCTCAGCCTCTT	GCCATAGAACTGATGAGAGGGAG
IL-1β	GAAATGCCACCTTTTGACAGTG	TGGATGCTCTCATCAGGACAG

Supplementary Table S3. The IBDQ scores of active IBD and IBD in remission.

Item	IBD in remission n=30	Active IBD n=55	*P* value
Bowel Symptoms	60.50（56.50，64.25）	42.00（35.00，50.00）	＜0.001
Systemic Symptoms	27.00（24.75，29.00）	19.00（12.00，25.00）	＜0.001
Emotional Function	65.00（60.00，73.25）	59.00（49.00，67.00）	0.005
Social Function	29.50（23.00，33.50）	18.00（12.00，27.00）	＜0.001
Total Score	180.50（163.00，201.25）	137.00（112.00,157.00）	＜0.001

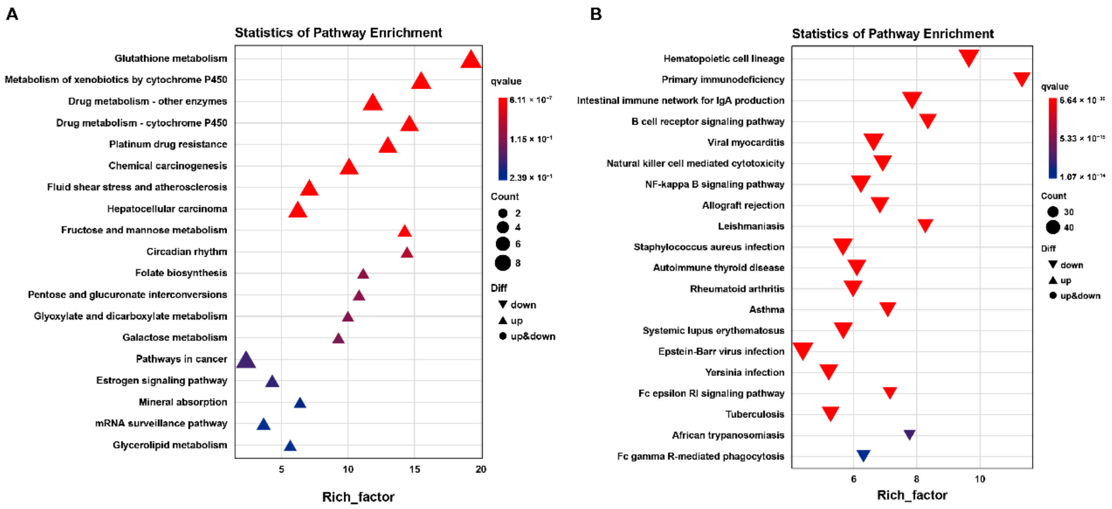

Supplementary Figure S1. KEGG pathway enrichment analysis of differentially expressed genes between the DSS and ERY+DSS groups. (**A**) KEGG pathways significantly enriched among genes upregulated in the ERY+DSS group relative to the DSS group; (**B**) KEGG pathways significantly enriched among genes downregulated in the ERY+DSS group relative to the DSS group.
